# Chemoenzymatic Kinetic resolution of (*R*)-malathion in aqueous media

**DOI:** 10.1186/s13065-015-0119-y

**Published:** 2015-09-09

**Authors:** Carlos A. Enríquez-Núñez, Alejandro A. Camacho-Dávila, Víctor H. Ramos-Sánchez, Gerardo Zaragoza-Galán, Lourdes Ballinas-Casarrubias, David Chávez-Flores

**Affiliations:** Facultad de Ciencias Químicas, Universidad Autónoma de Chihuahua, Circuito No.1 Campus Universitario, Chihuahua, Arboledas, Chihuahua 31125 Apartado Postal 669, , México

**Keywords:** Enantiomer, Enzymatic, Resolution, Malathion

## Abstract

**Background:**

Malathion (*R,S*)-diethyl-2-[(dimethoxyphosphorothioyl)sulfanyl]butanedioate is a chiral organophosphorus compound used widely as pesticide for suppression of harmful insects such as mosquitoes. It is well known that in biological systems (*R*)-malathion is the active enantiomer, therefore a sustainable approach could be the use of only the biologically active enantiomer. The resolution of the commercial racemic mixture to obtain the pure active enantiomer combined with a recycling of the undesired enantiomer through a racemization process could be an attractive alternative to reduce the environmental impact of this pesticide. Thus, this work evaluates the use of four commercially available lipases for enantioselective hydrolysis and separation of malathion enantiomers from the commercial racemic mixture.

**Results:**

Several lipases were methodologically assessed, considering parameters such as enzyme concentration, temperature and reaction rates. Among them, *Candida rugosa* lipase exhibited the best performance, in terms of enantioselectivity, *E* = 185 (selective to the (*S*)-enantiomer). In this way, the desired unreacted (*R*)-enantiomer was recovered in a 49.42 % yield with an enantiomeric excess of 87 %. The monohydrolized (*S*)-enantiomer was recovered and racemized in basic media, followed by esterification to obtain the racemic malathion, which was recycled. In this way, an enantioenriched mixture of (*R*)-malathion was obtained with a conversion of 65.80 % considering the recycled (*S*)-enantiomer.

**Conclusion:**

This work demonstrated the feasibility of exploiting *Candida rugosa* lipase to kinetically resolve racemic malathion through an environmentally friendly recycling of the undesired (*S*)-enantiomer.

**Graphical Abstract:**
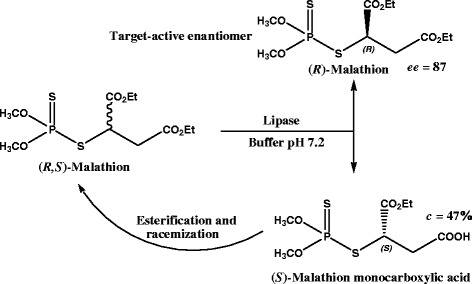
Lipase catalyzed enantioselective resolution of (R)-malathion in aqueous solvent.

## Background

The importance of molecular chirality has been widely recognized in life sciences due to the different activity of stereoisomers in biological systems. Chiral enantiomers have the same chemical and physical properties in achiral environments, but they are often markedly different in terms of their biological activity such as, toxicity and environmental fate in chiral environments [1–3]. Organophosphorus pesticides (OP) are among the most important chemicals used for protection against agricultural and household pests. It is estimated that OP are worth nearly 40 % of the global market and they are expected to prevail in the near future. Chiral pesticides currently account for about 33 % of the worldwide commercially available pesticides, including some chiral OP. Among these, malathion (*R,S*)-[diethyl 2-[(dimethoxyphosphorothioyl)sulfanyl]butanedioate] is one of the chiral OP extensively used for insect and pests control on grains, fruits, nuts, cotton, and tobacco; which indeed is commercialized as a racemic mixture: (*R,S*)-malathion [4]. It has been demonstrated that (*R*)-malathion is the target-active enantiomer of this particular racemic mixture. As a matter of fact, the (*R*)-enantiomer is 65x more toxic than the (*S*)-enantiomer [5, 6]. Therefore, the use of a single enantiomer could result in a reduction of the amount applied to treat pests minimizing the environmental impact and the cost of use. As the physical and chemical properties of enantiomers are the same, the preparation of pure enantiomers is still a challenge, especially in industrial processes. Generally, the industrial synthesis of chiral compounds generates the final products as racemic mixtures, which are difficult to separate. As an alternative to obtain pure enantiomers from racemic mixtures, the uses of enzymes is an attractive option.

Numerous biological processes are regulated by enzymes. In the last two decades, exploitation of enzymes in synthetic organic chemistry increased significantly due to its potential to catalyze reactions of specific substrates with high enantioselectivity and stereospecificity [7]. Lipases are biocatalysts extracted in low yields from animals and plants. They can also be obtained in higher yields by gene expression in an appropriate natural or recombinant microorganism. Lipases are the most used enzymes in synthetic organic chemistry It has been demonstrated that they possess a great versatility in catalysis of different reactions such as hydrolysis, esterification, transesterification and aminolysis [8–11].

Several lipases have been used for the enantiomeric resolution of alpha substituted carboxylic acids as ibuprofen, naproxen, ketoprofen, flurbiprofen, suprofen and other alpha and beta substituted carboxylic acids [12–14]. Recently, we reported an enantioselective hydrolysis of (*S*)-ibuprofen alkyl esters in aqueous media, also an enantioselective esterification in aqueous media with different alcohol moieties using lipases as biocatalysts [14, 15].

**Fig. 1:**
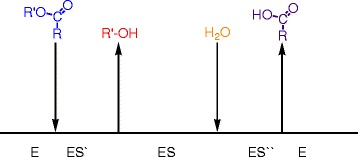
The ping-pong bi-bi scheme mechanism

Ideally, to obtain the pure (*R*)-malathion enantiomer, a lipase that selectively catalyzes the transformation of the (*S*)-enantiomer should be used. Thus, the separation of unreacted (*R*)-enantiomer could be performed easily by a simple extraction process. Some of the most widely used lipases for the enantioselective hydrolysis of (*S*)-enantiomers include *Candida rugosa* lipase, *Candida antarctica* lipase type B, Porcine pancreatic lipase and *Mucor javanicus* lipase [12, 14, 16]. In this work, the above mentioned lipases were used as biocatalysts for the enantioselective hydrolysis of (*S*)-malathion using commercially available (*R,S*)-malathion as reagent. It is widely accepted that lipase-catalyzed reactions (hydrolysis, esterification, alcoholysis, etc.) can be described by the *ping-pong bi-bi* mechanism, which proceeds by a nucleophilic attack on the carbonyl group promoted by a serine, histidine and an aspartate residues (also referred as “catalytic triad”), the resulting “acyl enzyme” intermediate can forward react with a nucleophile, such as water, alcohols or amines, regenerating the enzyme as shown in Fig. 1 [17].

The equilibrium between hydrolysis and synthesis depends highly on the water content in the reaction medium. Hydrolysis and ester synthesis are promoted by macro and micro aqueous solvent systems, respectively [18]. The aim of this work was to find the best lipase-type enzyme for enantioselective hydrolysis of (*R,S*)-malathion in aqueous media, in order to obtain the unreacted (*R*)-enantiomer and the monohydrolized (*S*)-enantiomer. These two were separated from each other and the latter was subsequently racemized in basic media and then esterified to recover a racemic mixture, which was indeed recycled, thus improving the efficiency of the overall process. The exploitation of pure or enriched (*R*)-enantiomer could improve the efficiency of this pesticide by minimizing its dose and ultimately its environmental impact.

## Results and discussion

### Enzyme assay

The hydrolytic activity of the four lipases was determined by a modified methodology previously developed [19]. Sunflower oil was used as enzymatic substrate, where *Candida rugosa* lipase showed the highest activity (31.8 U g^−1^ of biocatalyst), followed by porcine pancreatic lipase (13.3 U g^−1^ of biocatalyst), *Mucor javanicus* lipase (5.6 U g^−1^ of biocatalyst) and *Candida antarctica* lipase type B “Novozym 435” (3.6 U g^−1^ of biocatalyst). It is important to emphasize that the worst biocatalyst for this reaction was *Candida antarctica* lipase type B “Novozym 435”; despite being reported as a good biocatalyst for esterification and transesterification reactions. However, in this context many aspects might influence its biocatalyst activity. Perhaps, different enzyme sources (microorganism and mammalian vital organ) should naturally be expected to exhibit structural differences, which influence strongly on biocatalysts properties and activities, even in similar solvents [20]. In addition, the nature of the support and its polarity can also affect the enzyme conformation, as well as the partition of substrates and products from the enzyme environment, which might prevent the access of the substrate to the enzyme active site. On this research, *Candida antarctica* lipase type B immobilized in a macroporous acrylic resin was unable to catalyze the hydrolysis of sunflower oil and malathion, which is likely due to the relative hydrophobic surface of the resin that difficult the interaction between the enzyme active site and the substrate when the reaction occurs in an aqueous solvent [21].

### Lipase-catalyzed enantioselective hydrolysis of racemic malathion

**Fig. 2:**
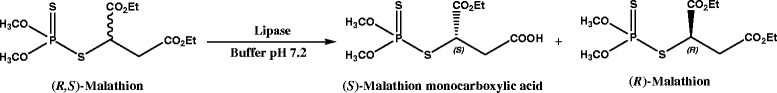
Lipase catalyzed enantiomeric resolution of racemic malathion

Based on the preliminary experiments described above, and in order to resolve the desired (*R*)-enantiomer from the racemic mixture, an enantioselective hydrolysis of (*R,S*)-malathion into (*S*)-malathion monocarboxylic acid and (*R*)-malathion was evaluated using *Mucor javanicus* Lipase, *Candida rugosa* Lipase and Porcine pancreatic Lipase as biocatalysts (Fig. 2). *Candida antarctica* Lipase type B (Novozym 435) was discarded because of its lack of activity during the preliminary hydrolysis essay. We consider that lipases attack first the less hindered ester group at position four farthest from the beta thioether substituent. The occurrence of a dicarboxylic acid resulting from hydrolysis at the two ester groups was confirmed by chiral HPLC chromatograms [22]. Only when the enzymatic reaction is left for more than 60 h, degradation products were evidenced on the chiral HPLC chromatogram showing new signals at retention times 9.673 min, 7.954 and 6.342 min [16].

**Fig. 3:**
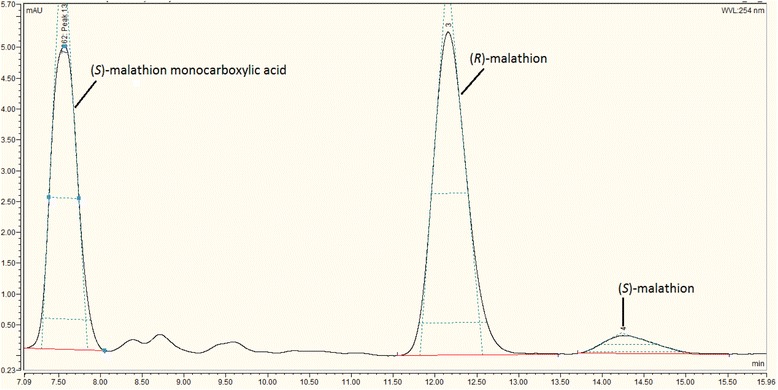
Chromatogram of the end of the enantioselective hydrolysis. (*S*)-malathion monocarboxylic acid (tR = 7.897 min), (*R*)-malathion (tR = 11.332 min), (*S*)-malathion (tR =12.984 min)

At the optimal reaction conditions (40 °C, 250 rpm, 0.15:1 w:w ratio of enzyme:substrate and phosphate buffer pH 7.2 as solvent) *Candida rugosa* lipase preferentially hydrolyze (*S*)-malathion into (*S*)-malathion monocarboxylic acid allowing to recover (*R*)-malathion in a satisfactory conversion yield (49.42 %) with a enantiomeric excess and enantioselectivity, of 87 and 185 respectively (Fig. 3). This was an obvious higher value than that obtained for Porcine pancreatic lipase or *Mucor javanicus* lipase, (Table 1). However, as it was expected, their performances were highly dependent on temperature and enzyme concentration. In fact, the best temperature was 40 °C for all reactions with enzyme concentrations 0–15 %, based on the substrate weight. Thus, when (10 mmol, 3.3 g) of racemic substrate reacted, followed by separation, extraction and evaporation at reduced pressure, in average 1.42 g of (*S*)-malathion monocarboxylic acid were recovered. This amount is equivalent to 4.70 mmol a ***c*** = 47.00 %. This agrees in a 98.86 % with the conversion values obtained from the chiral HPLC chromatograms areas, where the conversion determined was 47.54 %. The conversion and enantioselectivity values for reactions catalyzed by Porcine pancreas and *Mucor javanicus* lipases were very low, probably due to the poor dispersion of the enzyme on the reaction media or by other factors already mentioned. Also the nonpolar solvent dependency of *Candida antarctica* lipase type B was confirmed due to the low activity at conditions investigated. Previous reports state that *Candida antarctica* lipase type B is denatured in aqueous solvent above 40 °C. Whereas in dry media, it can withstand temperatures above 100 °C for extended time [22–24]. As expected, the hydrolysis of racemic malathion in aqueous media using *Candida antarctica* lipase type B did not occur within a range of temperature of 30 to 60 °C. Considering the hydrophobicity of the macroporous acrylic resin and the aqueous solvent on the reactions, we conclude that the substrate could not reach the enzyme active site.

**Table 1:** Lipase-catalyzed enantioselective hydrolysis of racemic malathion at optimal conditions

Enzyme % - Temp °C	*Candida rugosa* lipase			*Mucor javanicus* lipase			Porcine pancreatic lipase		
	*C*	*ee*_*p*_	*E*	*c*	*ee*_*p*_	*E*	*c*	*ee*_*p*_	*E*
5-30	11.2	45.5	28	1.4	25.9	12	1.3	63.4	77
5-40	23.8	47.8	32	2.8	27.1	13	4.8	70.7	59
5-50	26.3	38.5	31	3.2	23.4	10	9.7	60.2	32
5-60	25.3	2.5	17	2.9	13.0	5	7.8	42.3	21
10-30	16.8	37.5	24	1.87	43.4	16	2.1	70.2	32
10-40	34.5	47.1	55	2.56	45.9	116	8.8	72.1	68
10-50	37.2	41.2	30	2.83	45.1	28	8.8	56.2	52
10-60	33.8	30.4	20	2.04	43.3	22	9.0	41.1	32
15-30	35.2	32.3	80	2.12	65.7	80	9.7	56.43	62
15-40	46.5	86.8	185	4.88	72.9	94	12.88	58.61	81
15-50	47.1	54.2	83	4.9	70.4	108	13.12	45.67	62
15-60	48.4	32.4	23	4.4	67.8	86	13.36	40.71	24

Experimental condition: phosphates buffer pH 7.2 as solvent, 40 °C, stirring at 250 rpm, reaction time 48 h, 10 mmol of racemic malathion containing 40 mL of sodium phosphate buffer at pH 7.2. Enzyme concentration was 10 % of the substrate mass, conversion, *c*, enantiomeric excess, *ee*_*p,*_of desire product (*R*)-malathion and enantioselectivity, *E*

Our temperature effect studies aided to establish that temperatures above 40 °C leads to higher conversion yields with a lower enantioselectivity. The optimal temperature for the kinetic resolution of racemic malathion was determined to be 40 °C with all lipases. Although several studies of lipase kinetics have been carried out [21], the most common procedure is the use of the pseudo-first order model [25]. Due to the absence of the correspondent UV signal in the chiral HPLC chromatograms of the (*R*)-malathion monocarboxylic acid, it can be assumed that only (*S*)-malathion was hydrolyzed by lipases. Under this hypothesis, using reaction data obtained from the experiments, different kinetics were tested for the hydrolysis reactions of (*R,S*)-malathion under the optimal conditions (40 °C, barometric pressure and 250 rpm of agitation) with the 3 active enzymes and all reactions fitted to the pseudo first-order kinetics [26].

### Separation of enzymatic reaction products

**Fig. 4:**
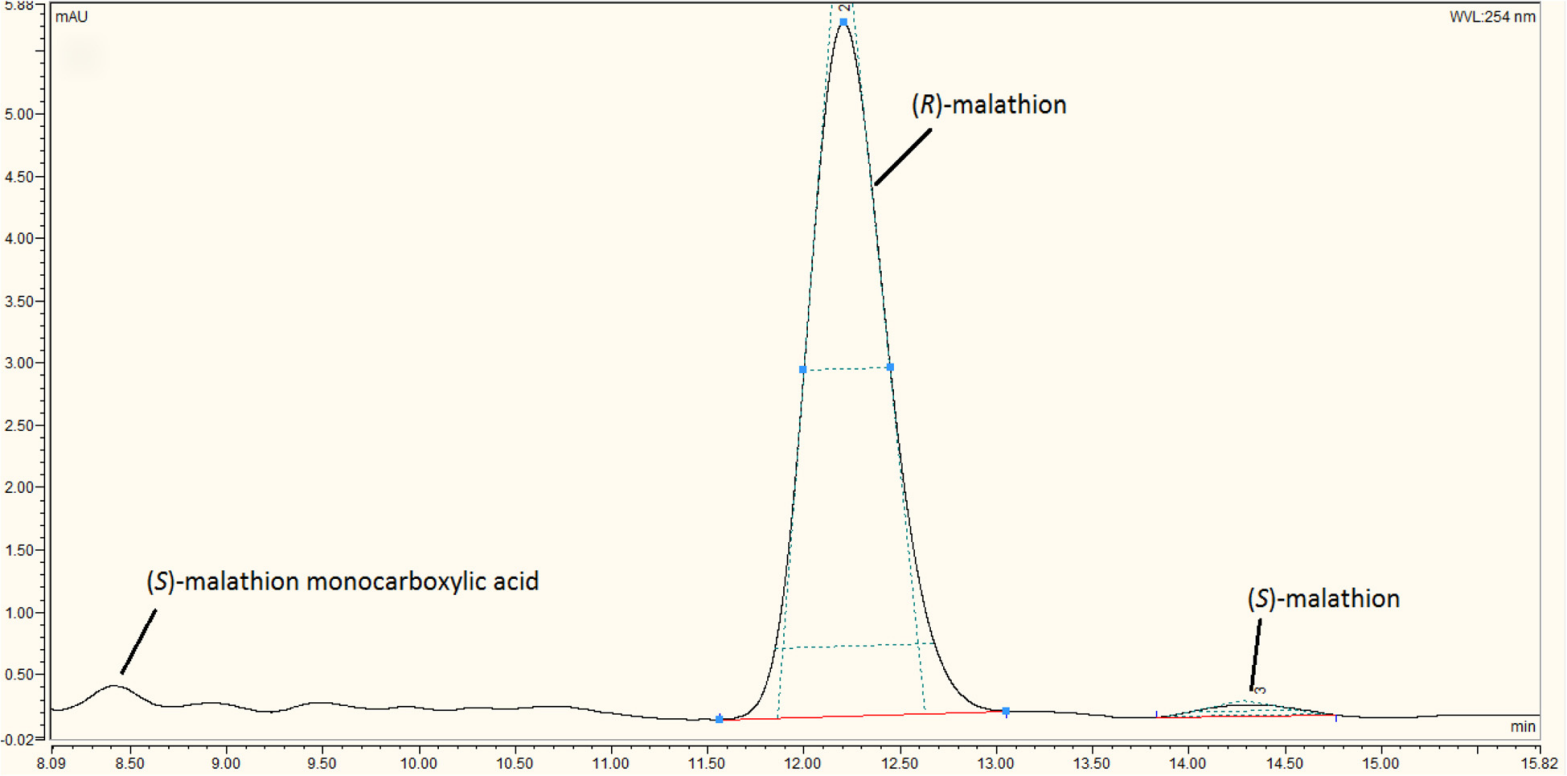
Chromatogram of the isolated (*R*)-malathion. (*R*)-malathion (tR = 12.984 min)

The undesired (*S*)-malathion monocarboxylic acid was separated from the unreacted (*R*)-malathion by extraction with a basic aqueous solution. It was noticed that the use of NaOH solutions caused the racemization of both the (*R*)-malathion and the (*S*)-malathion monoester. To prevent this, a 5 % aqueous solution of the less basic NaHCO3 was used. instead. The desired (*R*)-malathion was then extracted with hexanes and evaporated at reduced pressure. The product was analyzed by chiral HPLC (Fig. 4), polarimetry and NMR. The specific rotation of (*R*)-malathion was determined to be $\left[\alpha \right]\frac{25\;{}^{\circ}C}{D}=+76.92{}^{\circ},$ in 96%ethanol, indicating an ***ee*** of at least 86 %, in good agreement with the literature [27], and matching closely with the ***ee*** = 86.7 calculated using the chiral HPLC areas collected. These results are in contrast to mammalian based hydrolyses where it has been demonstrated that (*R*)-malathion undergoes more rapid degradation in the environment than the (*S*)-enantiomer [28–30].

### Racemization of (*S*)-malathion monocarboxylic acid

**Fig. 5:**
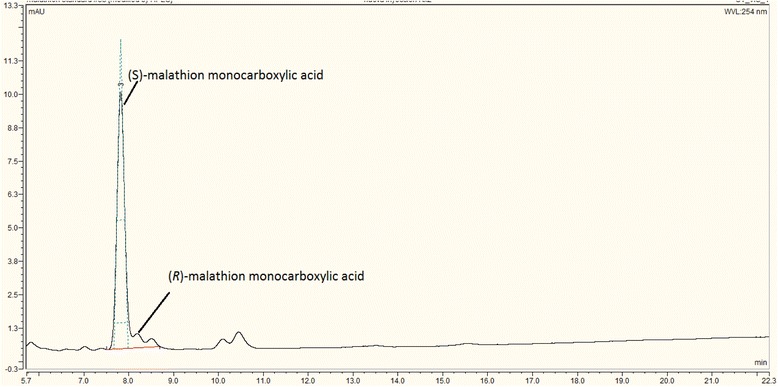
Chromatogram of the isolated (*S*)-malathion monocarboxylic acid. (*S*)-malathion monocarboxylic acid (tR = 7.897 min)

**Fig. 6:**
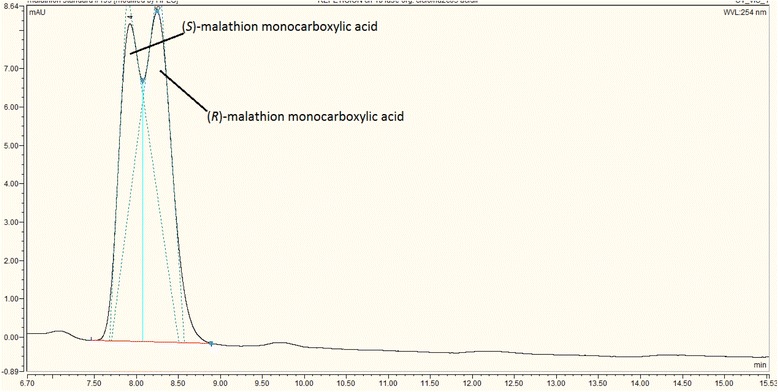
Racemic malathion monocarboxylic acid. (*S*)-malathion monocarboxylic acid (tR = 7.897 min) and (*R*)-malathion monocarboxylic acid (tR = 8.436 min)

Once the (*R*)-malathion was isolated and characterized, the next goal was to racemize the undesired (*S*)-enantiomer (Fig. 5). To carry out the racemization, aqueous Na2CO3 solution was selected as base. It was found that when concentrations higher than 30 % of Na2CO3 were used, the reaction took place at higher rates but resulting in a racemization and extensive hydrolysis of the thiophosphonte ester bond. The optimal concentration was found to be at 10 % Na2CO3. Thus, after the racemization, acidification and extraction with hexanes provided the racemized product. Thus when 5 mmol (1.65 g) of (*S*)-malathion monocarboxylic were used for racemization, 1.58 g of racemic malathion monocarboxylic acid (4.78 mmol) were recovered and confirmed by chiral HPLC (Fig. 6). The final and desired product, (*R*)-malathion, was then was characterized by chiral HPLC, polarimetry and FTIR which are in agreement with the literature.

### Esterification of (*R,S*)-malathion monocarboxylic acid

**Fig. 7:**
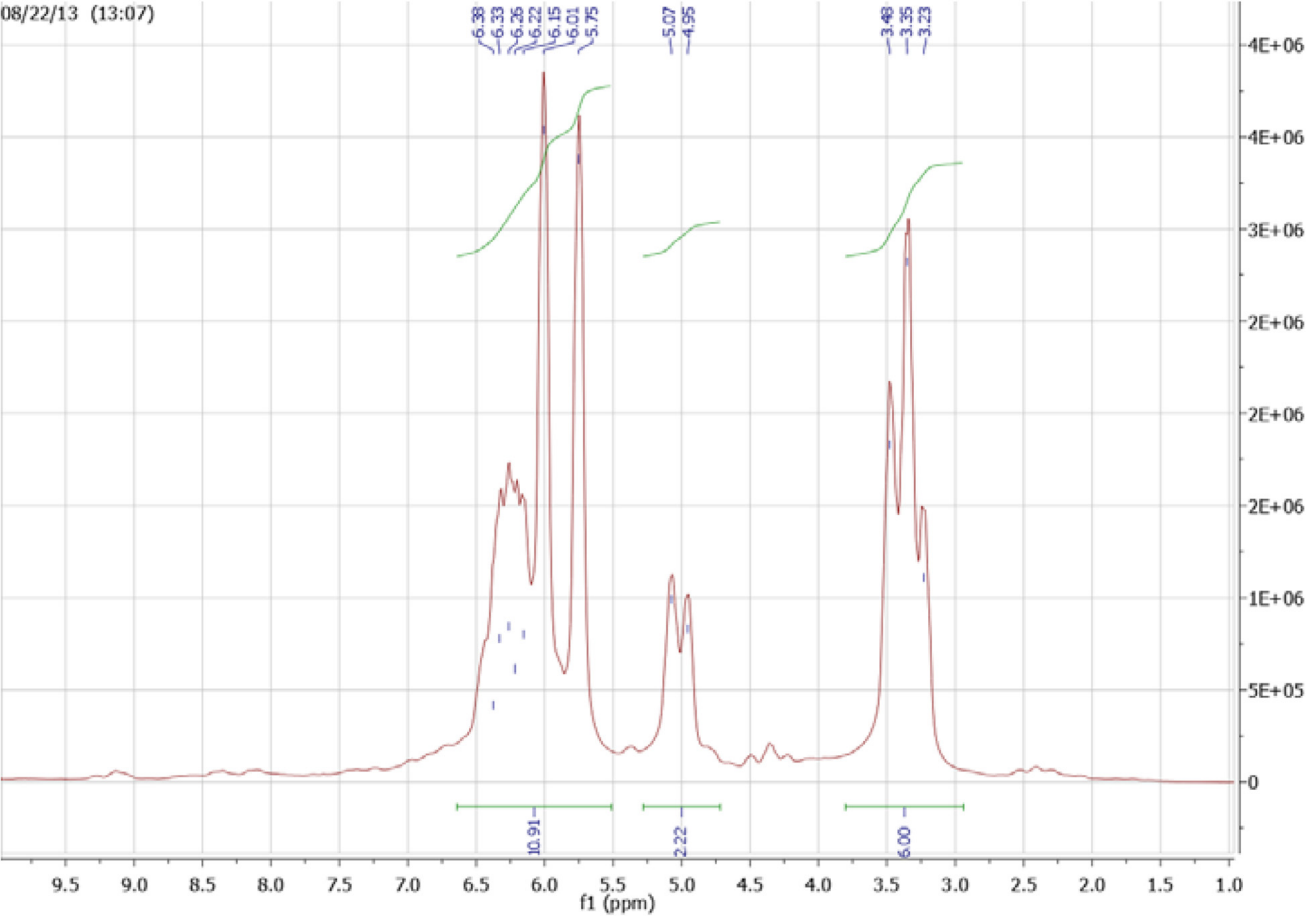
^1^H NMR spectrum of malathion

Once the (*R,S*)-malathion monocarboxylic acid as recovered, it was submitted to an esterification reaction to obtain the racemic malathion. This was carried out using excess of ethanol with a catalytic amount of H_2_SO_4_ in a Dean-Stark trap containing molecular sieves to remove the formed water. In this way a conversion of 80.27 % yield was obtained. The obtaines racemic malathion was characterized by HPLC, IR and NMR (Fig. 7).

## Experimental section

Too ensure the reproducibility and accuracy of the data each experiment was conducted in triplicate. Results reported are the average values of the experiments.

### Materials

Racemic malathion (*R,S*)-diethyl 2-[(dimethoxyphosphorothioyl)sulfanyl] butanedioate was isolated from inexpensive consumer pesticide “Malathion 1000®” donated by Velsimex S.A. de C.V. *Candida rugosa* lipase, *Candida antarctica* lipase type B, Lipase from Porcine pancreas and *Mucor javanicus* lipase were obtained from Sigma Aldrich Company. HPLC grade hexanes, isopropanol, sodium phosphate monobasic and dibasic were purchased from MAESA Chemicals. Cyclohexane, ethyl acetate and ethanol were purchased from FERMONT Company. All other analytical grade reagents and solvents were obtained from commercially sources.

### High performance liquid chromatography (chiral) analysis

In order to monitor the development of the enantioselective enzymatic hydrolysis, the product separation and the acid catalyzed esterification reaction, high performance liquid chromatography was performed with chirlacel OJ chiral column (Diacel Chemical Industries). The HPLC instrument was equipped with a Dionex LPG-3400-D Quaternary Analytical Pump, Dionex UltiMate 3000 Diode Array Detector, Dionex solvent degaser and Chromeleon CM-PCS-1 Software. The mobile phase normally used was hexanes/isopropanol/trifluoroacetic acid (95:4.9,0.1 % v/v/v). The UV detector wavelength was set to 254 nm, the flow rate was 1.0 mL/min and the temperatures of the column and injection compartments were 15 °C. The chromatographic signal peaks of racemic malathion were confirmed by comparing their retention times (11.332 min (*R*)-malathion, 12.984 min for (*S*)-malathion and 7.897 min (*S*)-malathion monocarboxylic acid) and UV spectra with the obtained with the reference standard. The optical rotation was measured using an Atago® POLAX 2 L semiautomatic Polarimeter at 22 °C with a sodium lamp at 589 nm using samples with concentrations of 1 g/100 mL in anhydrous ethanol. Typically, about a gram of the mixture was separated and analyzed per run. A BÜCHI® rotary evaporator (Model R-210) was used to remove volatile solvents under reduced pressure. A Perkin Elmer Fourier Transform Infrared Spectrometer Model IRGX with Attenuated Total Reflection sampler was used for the characterization.

### Isolation of (*R,S*)-malathion from commercially available pesticide formulation

Using 200 mL of cyclohexane as mobile phase in a 2.5 × 15 cm flash chromatography column packed with 5 μm silica gel as stationary phase, racemic malathion was isolated from a commercially available malathion pesticide formulation. The mobile phase with the extracted racemic malathion was evaporated at reduced pressure to isolate the racemic malathion. The isolated material was analyzed by chiral HPLC, H^1^ NMR and FTIR.

### Lipase catalyzed enantioselective hydrolysis of malathion

In a typical reaction, 3.30 g (10 mmol) of racemic malathion, 40 mL of 0.1 M sodium phosphate buffer pH 7.02, 0.165–0.66 g of lipase and 0.5 g of Celite 577 fine for dispersion of lipase particles were added in to a 100 mL dry baffled-flat-bottom flask. The reactions were stirred at 300 rpm and 40 °C. The mixture was analyzed by chiral HPLC before adding lipase. By taking 1 mL samples and using extraction with hexanes, the reactions were monitored for at least 48 h by chiral HPLC and then stopped and centrifuged at 4500 rpm for 6 min for the enzyme recovery by decanting the reaction solution. The remaining oil was weighed and saved for further separation and analysis.

### Enantioselectivity value (*E*-value) measurements

The value of enantioselectivity (***E***) was calculated from the enantiomeric excess of the substrate (***ee***) and the conversion degree (***c***) according to the equations 1 and 2 described by Chen et al. [23].

1 $$ \boldsymbol{C}=\frac{\boldsymbol{e}{\boldsymbol{e}}_{\boldsymbol{s}}}{\boldsymbol{e}{\boldsymbol{e}}_{\boldsymbol{s}}+\boldsymbol{e}{\boldsymbol{e}}_{\boldsymbol{p}}} $$

2 $$ \boldsymbol{E}=\frac{\boldsymbol{ln}\;\Big[\left(\boldsymbol{1}\boldsymbol{\hbox{-}}\boldsymbol{c}\right)\left(\boldsymbol{1}\boldsymbol{\hbox{-}}\boldsymbol{e}{\boldsymbol{e}}_{\boldsymbol{s}}\right)}{\boldsymbol{ln}\;\Big[\left(\boldsymbol{1}\boldsymbol{\hbox{-}}\boldsymbol{c}\right)\left(\boldsymbol{1}\boldsymbol{\hbox{-}}\boldsymbol{e}{\boldsymbol{e}}_{\boldsymbol{s}}\right)} $$

### Isolation of (*R*)-malathion and racemization of (*S*)-malathion monocarboxylic acid

In order to isolate the desired product (*R*)-malathion and to avoid its racemization, a weak base was used to produce the (*S*)-malathion sodium monocarboxylate. About 40 mL of the decanted reaction solution was extracted three times with 40 mL of 5 % v/w NaHCO_3_ aqueous solution. The desired (*R*)-malathion was isolated by removing the solvent by evaporation at reduced pressure.

### Racemization of (*S*)-malathion monocarboxylic acid

In a 100 mL round bottom flask the undesired subproduct (*S*)-malathion monocarboxylic acid 1.51 g (5 mmol) was mixed with 40 mL of aqueous Ca_2_CO_3_ at different concentrations 5, 10, 15, 20, 25 and 30 % w/w. Then the mixture was stirred for 60 min. In order to monitor the racemization reactions, 1 mL of sample was removed from the reaction solution to slowly be acidified with 3 % HCl w/v to induce the formation of the corresponding malathion monocarboxylic acid. Once the racemization was achieved, the solution was acidified and the racemic mixture was extracted by triplicated with hexanes and evaporated at reduced pressure to be analyzed by chiral HPLC determining the residual weight and enantiomeric excess.

### Esterification of (*R*,*S*)-malathion monocarboxylic acid

Typically in a 100 mL baffled-flat-bottom flask 1.51 g (5 mmol) of (*R,S*)-malathion monocarboxylic acid, 5 mL of ethanol and 30 mL of solvent (anhydrous ethanol, acetone, dioxane and acetonitrile) and 1 mL of concentrated sulfuric acid were added. The mixture was refluxed and stirred for 8 h using a Dean Stark apparatus and molecular sieves to remove water, the undesired byproduct. The reaction mixture was extracted by triplicated with 30 mL of cyclohexane. The combined organic layers were dried with magnesium sulfate and evaporated at reduced pressure to give (*R,S*)-malathion. The compound matched perfectly with the physical characteristics of malathion, an oily compound with garlic odor and yellow color. The isolated malathion was analyzed by Chiral HPLC and the optical rotation matching satisfactorily with the standard of reference.

## Conclusion

Comparison of commercially available lipases as biocatalysts for the enantioselective hydrolysis of (*R,S*)-malathion was performed. the obtained results suggested that *Candida rugosa* lipase was the best biocatalyst of the lipases studied, showing conversion yields up to 49.47 %, with acceptable enantioselectivity of *E* = 185 to the *S* enantiomer. It was found that temperature of 40 °C and a weight ratio of 0.15:10 for substrate:enzyme were optimal for all reactions. The monohydrolyzed (*S*)-malathion, was successfully racemized and esterified obtaining, (*R,S*)-malathion for further recycling. In conclusion, this work demonstrated the feasibility of exploiting *Candida rugosa* lipase to kinetically resolve racemic malathion through an environmentally friendly recovery of the undesired (*S*)-enantiomer. This methodology could also be applied for the resolution of other OP’s such as Phentoate, which also contains a chiral group and it is indeed a pesticide widely used worldwide.
